# Short-term mental distress in research participants after receiving cardiovascular risk information

**DOI:** 10.1371/journal.pone.0217247

**Published:** 2019-05-24

**Authors:** Åsa Grauman, Mats G. Hansson, Arvid Puranen, Stefan James, Jorien Veldwijk

**Affiliations:** 1 Centre for Research Ethics & Bioethics, Uppsala University, Uppsala, Sweden; 2 Sunderby Hospital, Region Norrbotten, Luleå, Sweden; 3 Department of Medical Sciences, Cardiology, Uppsala University, Uppsala, Sweden; 4 Erasmus School of Health Policy & Management, Erasmus University, Rotterdam, Netherlands; 5 Erasmus Choice Modelling Centre, Erasmus University, Rotterdam, Netherlands; Medizinische Universitat Graz, AUSTRIA

## Abstract

**Background:**

Understanding of how cardiovascular risk information influence individuals is critical for the practice of risk assessment and the management of patients with cardiovascular disease.

**Objectives:**

The objective of this study was to investigate change in mental distress among research participants after undergoing a cardiovascular risk assessment and receiving individual test results.

**Methods:**

In 2017, a questionnaire measuring mental distress after taking part in a risk assessment was distributed among 615 participants in the Swedish Cardiopulmonary Bio Image Study in Uppsala, Sweden, aged 50–64 years. Outcome measures were re-assessed after three months (30% were lost to follow-up).

**Results:**

There were no differences in outcomes after three months for participants with normal test results or for participants who were referred to primary health care. Mental distress increased in participants who were referred to the hospital, and were further explained by the fact that these participants were diagnosed with coronary artery stenosis.

**Conclusions:**

CV risk information can be provided to individuals with lower levels of risk without concerns of inducing mental distress. However, in order to prevent unnecessary worry in contexts similar to this study, one should be prepared for different risk outcomes and plan for support for individuals with higher risk. The increased utility of powerful, yet not fully mature, imaging techniques requires careful considerations extending beyond medical risks and benefits; the clinician must also take into account the risk of mental distress and secure support when necessary.

## Introduction

The Swedish CArdioPulmonary BioImage Study (SCAPIS) was initiated to predict and prevent cardiovascular diseases (CVD) and chronic obstructive pulmonary disease (COPD). The study includes thorough health examinations of 30,000 Swedish men and women aged 50–64 years. After conducting the health examinations, the participants receive their personal test results including several CV risk factors. As seen in the SCAPIS pilot study, the baseline examinations of study participants will identify a substantial proportion of individuals with specific risk factors for CVD [1]. The current study was initiated following reports of individual participants expressing concern about receiving CV risk information and how it affected them. On the one hand, information was considered as positive and proactive; on the other hand, participants expressed concerns that the information may negatively affect their quality of life.

Psychosocial stress, including depression and anxiety, is itself a strong risk factor for cardiovascular diseases [2]. A high level of distress can also cause individuals to avoid further checkups [3] and interfere with individuals’ cognitive capacity and thus reduce their ability to process information [4]. It is therefore important to monitor the effects of risk assessment and risk information on the psychological well-being of individuals.

Previous research is inconclusive regarding the emotional impact of CV risk assessment. Disclosure of negative test results has caused short-term psychological distress [5, 6] and some studies indicate that being labeled with e.g. hypertension can have a negative impact on individuals’ quality of life [7]. Other studies report no such effects [8–12].

Risk assessments in clinical practice have the potential to prevent cardiovascular diseases [13], and are therefore recommended by the European Guidelines on Cardiovascular Disease Prevention in Clinical Practice [14]. Patients need to be appropriately informed about their disease and risk but there is a risk that information increases stress that may negatively affect outcomes further. A better understanding of risk information and how that affects the individual and risk in itself is critical in the management of patients with cardiovascular disease.

Furthermore, it is also important to see whether large-scale preventive testing might cause more psychological distress than preventive potential.

The objective of this study was to investigate change in mental distress among research participants after undergoing a cardiovascular risk assessment and receiving individual test results.

## Material and methods

### 2.1 Design and study population

The health examinations in SCAPIS were comprehensive and included computed tomography (CT) including coronary angiography, high-resolution ultrasound, clinical measurements, anthropometry, blood sampling and questionnaires. After completing the baseline examinations, all participants received a written report of some of the test results e.g. waist circumstance, Body Mass Index (BMI), blood pressure and heart rate. In their electronically accessible health records, the participants could find test results regarding the result for coronary artery imaging, cholesterol and glucose levels. In the case of clinically relevant findings, participants were referred to either a primary health care center (PHCC) or specialized care, where they received routine care [1]. For information about Patient Accessible Electronic Health Records in Sweden, see Hägglund & Scandurra 2017 [15].

**Fig 1:**
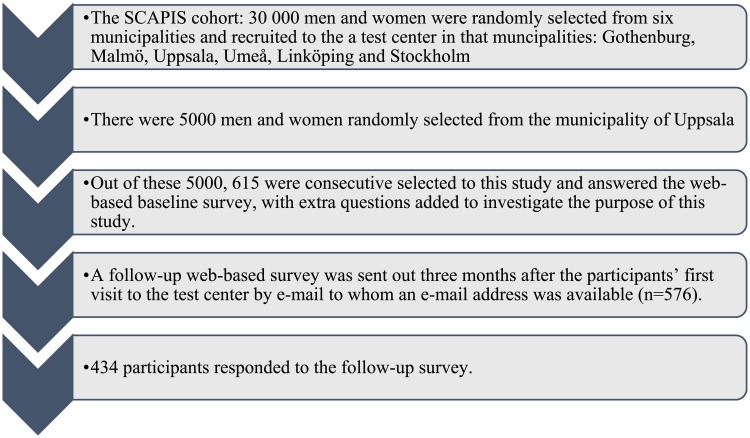
Study process.

The 30, 000 SCAPIS participants were invited to one of six study sites in Sweden. At the Uppsala study site, 5, 000 men and women, aged 50–64, were randomly selected from Uppsala Municipality. During the recruitment of these 5,000 individuals, extra questions were added to the SCAPIS questionnaire for the purpose of this study. After 615 participants had visited the test site and consecutively responded to the extended questionnaire, the extra questions were removed to not burden more participants than necessary, see Fig 1. The study was approved by the Regional Ethical Review Board of Uppsala (Reg. no. 2016/256).

The online baseline survey included questions about worry about experiencing a myocardial infarction, mental health, health literacy, numeracy and risk perception, which was added to the original survey in SCAPIS answered by all participants between their first and second visit. Three months after the 615 participants’ first visit to the test center, an online follow-up survey was sent e-mailed everyone who provided their e-mail address (n = 576). Two reminders were sent out at approximately two weeks intervals. In total 434 participants answered the follow-up questionnaire (response rate = 70%). Data collection took place from February—August 2017. Information about participants’ sociodemographic factors, life-style, perceived stress, health-related quality of life, family history of myocardial infarction and medical background was collected from the SCAPIS database.

### 2.2 Measures

#### 2.2.1 Mental distress

Worry about experiencing a myocardial infarction (“Are you worried about having a heart attack?”) was assessed on a 5-point scale ranging from not worried at all to extremely worried.

Health-related quality of life was assessed by Short form health survey (SF-12). The twelve items were combined into a mental component summary (MCS) and a physical component summary (PCS), each expressed by a value between 0–100, where 100 represents excellent health [16]. In three of the twelve questions, one of the alternatives for answering was missing. The weight from the missing alternatives was evenly distributed to the remaining alternatives for the specific question to compensate for the missing alternative.

Mental health was assessed by the Hospital Anxiety and Depression Scale (HADS). It consists of 14 items, which are divided into an anxiety subscale (HADS-A) and a depression subscale (HADS-D). The cut-off levels are: 0–7 normal, 8–10 borderline, 11–21 abnormal [17].

#### 2.2.2 Test results

To allow analysis of differences in mental distress due to the type of test result received, questions about referral to a PHCC or hospital due to findings in SCAPIS were included, as were questions related to diagnosis of hypertension, high cholesterol or coronary artery stenosis.

#### 2.2.3 Characteristics of participants

Sociodemographic variables included gender, age, education level (graduated from primary school, high school or university) and whether they were born in Sweden.

Health literacy (HL) was measured using the validated Swedish version of The Communicative and Critical Health Literacy scale (S—C & C HL scale). It has five-item, five-point Likert scale [18]. Participants who only answered ‘strongly agree’ or ‘agree’ were categorized as having sufficient HL; those who answered ‘strongly agree’, ‘agree’ or ‘partially agree’ were categorized as having problematic HL and those who answered ‘strongly disagree’ or ‘disagree’ on any item were categorized as having inadequate HL [19].

Numeracy was assessed by using the Short 3-item Version of Subjective Numeracy Scale, a three-item, five-point Likert scale, from which a mean summary score was calculated [20].

Self-perceived stress was dichotomized into low level of stress (included answers “never experienced stress”, “experienced some periods of stress” and “experienced a period of stress the last five years”) versus high level of stress (included answers “constant stress the last year” and “constant stress the last five years”).

Medical background was assessed by asking about treatment for or diagnosis of CVD, diabetes, hypertension or high cholesterol before participating in SCAPIS. The section also included questions about family history of myocardial infarction. Family included parents and siblings. Having multiple risk factors intended having more than one of the following risk factors: smoking, CVD, abdominal obesity, hypertension, diabetes, high cholesterol.

Smoking was dichotomized into daily smoking and occasional smoking versus stopped smoking and never smoked. Waist circumference was measured in centimeters and used to compute abdominal obesity, >87 cm for women and >101 cm for men.

### 2.3 Statistical analysis

Descriptive statistics were performed to describe the study population in terms of sociodemographic factors, medical background, life style, risk perception and type of test result.

Independent t-tests, one-way ANOVA and Pearson’s correlation were used to analyze differences in mental distress between different referral groups and descriptive variables at baseline.

To test for differences in mental distress between baseline and three months after risk assessment paired t-tests were conducted for all dependent variables: worry about experiencing a myocardial infarction, anxiety, depression, MCS and PCS. Besides total sample, the analyses were conducted separately for participants who were referred to either PHCC or hospital. Paired t-tests were also conducted separately for participants diagnosed with hypertension, high cholesterol or coronary artery stenosis.

Differences in mental distress between baseline and three months after risk assessment were further tested in multiple linear regression models. The follow-up measurements of mental distress were used as dependent variables and the referral group comprised the independent variables, adjusting for baseline measures of psychological factors, as well as for age and health literacy.

The variable for worry about experiencing a myocardial infarction was measured on an ordinal scale but treated as a numerical variable and tested with parametric tests. The result was confirmed with sensitivity analysis using a non-parametric test.

All statistical analyses were performed in SPSS statistical software version 4.0. All reported p values were two-tailed and statistical significance was defined as p < 0.05.

This work was funded by a grant from the Swedish Heart and Lung Association (grant number: 20150049).

## Results

Descriptive statistics of the participants in the follow-up sample (n = 434) are presented in Table 1, both in total and stratified by exposure.

**Table 1:** Characteristics of participants presented as total distribution and divided by exposure groups. Continuous variables are expressed as mean (SD), categorical as percentages.

N = 434		Total	Not referred	Referred toPrimary care	Referred to hospital
Age		58.0 (4.4)	57.7 (4.3)	58.3 (4.6)	59.1 (4.0)
Gender (*Female)*		53.0%	55.5%	49.4%	42.6%
Education	Primary school	6.7%	6.0%	5.7%	13.0%
	High school	43.1%	39.9%	50.6%	44.4%
	University	50.2%	54.1%	43.7%	42.6%
Born in Sweden	Yes	90.1%	87.2%	94.3%	98.1%
Health literacy	Sufficient	62.0%	66.4%	65.1%	57.7%
	Problematic	30.4%	30.4%	30.1%	36.5%
	In-adequate	3.5%	3.0%	4.8%	5.8%
Numeracy		3.9 (0.8)	3.9 (0.8)	3.8 (0.9)	3.8 (1.0)
Hypertension^a^		22.1%	20.4%	23.0%	31.5
High cholesterol^a^		11.3%	9.3%	8.0%	25.9%
Diabetes^a^		3.7%	3.2%	2.3%	7.4%
CVD^a^		6.0%	5.0%	6.9%	11.1%
Multiple risk factors	2	16.6%	15.2%	18.6%	20.4%
	3 or 4	8.4%	7.2%	5.8%	18.5%
Family history of myocardial infarction		24.7%	22.6%	31.0%	26.4%
General health	Bad	1.6%	1.4%	2.3%	1.9%
	Somewhat good	15.2%	12.8%	19.5%	16.7%
	Good	34.6%	33.5%	37.9%	37.0%
	Very good	35.0%	37.7%	26.4%	38.9%
	Excellent	13.6%	14.6%	13.8%	5.6%
Self-perceived stress	Low	81.3%	82.1%	79.3%	79.6%
	High	18.8%	17.9%	20.7%	20.4%
HADS Anxiety^b^	Borderline case	11.1%	9.6%	10.8%	17.3%
	Case	6.5%	7.4%	3.6%	3.8%
HADS Depression^b^	Borderline case	5.8%	5.5%	6.0%	3.8%
	Case	2.6%	3.3%	1.2%	1.9%
Abdominal obesity^c^		49.8%	45.2%	55.2%	61.1%
Smoker		7.0%	7.9%	8.1%	-

^a^ Treated or diagnosed before participating in SCAPIS

^b^According to the cutoffs for Hospital Anxiety and Depression Scale

^c^Waist circumference >87 cm for woman, >101 for men.

### 3.1 Psychological factors at baseline

The majority of the participants were either not at all worried (46.7%) or a bit worried (45.5%) about experiencing a myocardial infarction at baseline. Differences in worry were associated with general health, a family history of myocardial infarction, age, health literacy, abdominal obesity, stress and having multiple risk factors (p <0.01).

At baseline, participants referred to the hospital reported higher levels of worry about experiencing a myocardial infarction (p = 0.001) than other referral groups. Otherwise, there were no differences in psychological factors between referral groups at baseline.

### 3.2 Differences in psychological factors after receiving test results

The aim of this study was to investigate change in mental distress among research participants after undergoing a cardiovascular risk assessment and receiving individual test results. Twenty percent of participants were referred to PHCC and 14.3% to the hospital for specialized care. Seventeen participants reported being diagnosed with coronary artery stenosis, 19 with hypertension and 10 with high cholesterol. Eighty percent of the participants reported that they had looked at test result in their Patient Accessible Electronic Health Records.

To test for differences in mental distress between baseline and three months after risk assessment, paired t-tests were conducted for all outcome variables based on referral group. For participants who were not referred or referred to PHCC, no significant differences were found for any of the outcomes between baseline and after three months. For participants referred to the hospital there was an increase in worry and anxiety, and a decrease in MCS, which together indicate an increase in mental distress. See Table 2. Sensitivity analysis with a non-parametric test of worry produced the same result.

**Table 2:** Differences in psychological factors at baseline and after three months.

Psychological factors		N 434	Baseline	Three months
			Mean(SD)	Mean(SD)
Worry about experiencing a myocardial infarction	Total	408	1.6 (.7)	1.7 (.7)
	Not referred	266	1.6 (.7)	1.6 (.7)
	Referred PHCC	82	1.6 (.6)	1.7 (.6)
	Referred hospital*	52	1.8 (.7)	2.0 (.8)
SF-12 PCS	Total	418	50.7 (7.7)	50.5 (7.5)
	Not referred	271	50.9 (7.4)	50.9 (7.0)
	Referred PHCC	84	50.7 (8.3)	50.1 (8.0)
	Referred hospital	53	49.6 (8.5)	49.3 (7.5)
SF-12 MCS	Total	418	51.8 (9.1)	51.1 (10.3)
	Not referred	271	51.8 (9.0)	51.5 (9.6)
	Referred PHCC	84	52.0 (8.6)	51.5 (10.0)
	Referred hospital*	53	52.0 (9.1)	49.6(11.0)
HADS Anxiety score	Total	416	4.4 (3.5)	4.5 (3.6)
	Not referred	271	4.4 (3.6)	4.4 (3.6)
	Referred PHCC	83	4.0 (3.1)	4.0 (3.2)
	Referred hospital*	52	4.7 (3.1)	5.4 (3.4)
HADS Depression score	Total	416	3.3 (2.9)	3.3 (2.9)
	Not referred	271	3.3 (3.1)	3.2 (2.9)
	Referred PHCC	83	3.2 (2.6)	3.4 (2.7)
	Referred hospital	52	3.6 (2.4)	3.8 (2.9)

*P>0.05

**P>0.01

^A^N = 343

Data collected in Uppsala, Sweden, 2017.

Paired t-tests were also conducted for all outcome variables based on diagnosis with high cholesterol, hypertension or coronary artery stenosis. There were changes in worry, anxiety and MCS for participants diagnosed with coronary artery stenosis, one of the conditions for hospital referral. However, few participants were diagnosed with CV risk factors and the result should therefore be interpreted with caution.

Associations between change in mental distress after three months and referral groups after three months were tested in multiple linear regression models, adjusting for age and health literacy level. Diagnosis with coronary artery stenosis was also adjusted for, since this was one reason for referral to the hospital in SCAPIS, and because there was a significant change in the paired t-test analysis.

No associations between change in worry, anxiety and MCS and referral were observed in the regression models when adjusting for age, health literacy and diagnosis with coronary artery stenosis. There was an association between change in worry and diagnosis with coronary artery stenosis (p<0.000). There was also an association between change in anxiety and diagnosis with coronary artery stenosis (p = 0.03). No associations were observed for MCS, PCS or depression. The results from the regression models are presented in Table 3.

**Table 3:** Multiple linear regression analysis of differences between referral groups three months after risk assessment, adjusting for diagnosis of coronary artery stenosis, age and health literacy.

	Model 1 β	CI for Beta		Adj. R square	Model 2 β	CI for Beta		Adj. R square
		Lower	Upper			Lower	Upper	
**Worry after 3 months**				0.40				0.43
Worry at baseline	0.62**	0.56	0.72		0.64**	0.56	0.72	
Referral to hospital	0.10*	0,05	0,36		0.05	-0.06	0.26	
Referral to PHCC	0.01	-0.11	0.16		0.02	-0.13	0.14	
Coronary artery stenosis					0.15**	0.28	0.88	
Age					-0.03	-0.02	0.01	
Health literacy^A^					0.04	-0.05	0.18	
**MCS after 3 months**				0.38				0.39
MCS baseline	0.61**	0.58	0.75		0.61**	0.58	0.75	
Referral to hospital	-0.10*	-4.83	-0.60		-0.07	-4.31	0.16	
Referral to PHCC	-0.01	-2.02	1.71		0.00	-1.88	1.90	
Coronary artery stenosis					-0,08	-8.23	-0.28	
Age					0.04	-0.09	0.26	
Health literacy^A^					-0.06	-2.82	0.35	
**PCS after 3 months**				0.43				0.43
SF-12 P baseline	,654**	,547	,685		0.65**	0.55	0.68	
Referral to hospital	-,018	-1,878	1,131		0.000	-1,60	1,60	
Referral to PHCC	-,016	-1,609	1,040		-0.01	-1.52	1.14	
Coronary artery stenosis					-0.05	-4.99	1.10	
Age					-0.02	-0.15	0.10	
Health literacy^A^					-0.01	-1.37	0.92	
**HADS A after 3 months**				0.56				0.56
HADS A at baseline	0.72**	0.69	0.82		0.74**	0.69	0.82	
Referral to hospital	0.07*	0.03	1.33		0.04	-0.24	1.14	
Referral to PHCC	-0.03	-0.85	0.29		-0.03	-0.90	0.27	
Coronary artery stenosis					0.08*	0.14	2.71	
Age					-0.02	-0.07	0.03	
Health literacy^A^					0.02	-0.34	0.63	
**HADS D after 3 months**				0.54				0.54
HADS D at baseline	0.74**	.67	.80		0.73**	0.66	0.80	
Referral to hospital	0.05	-0.13	.94		0.05	-0.18	0.96	
Referral to PHCC	0.02	-0.36	0.58		0.02	-0.37	0.60	
Coronary artery stenosis					0.02	-0.82	1.32	
Age					-0.5	-0.08	0.01	
Health literacy^A^					0.02	-0.25	0.55	

*p<0.05

**p<0.01

Data from SCAPIS research participants. Men and woman 50–64 years old. Data collected in Uppsala, Sweden, 2017. N = 434

^A^Sufficient health literacy compared to problematic or in-adequate health literacy

The results from the regression models indicate that a change in worry and anxiety after referral to the hospital can be explained by the fact that a large share of participants referred to the hospital found out about coronary artery stenosis. However, it cannot explain the change in MCS after referral to the hospital.

## Discussion

Psychosocial stress, is a strong risk factor for cardiovascular diseases. Concerns about how receiving CV risk information would affect the research participants in SCAPIS was investigated in this study. Our results provide evidence that these concerns are not substantiated for those with lower levels of risk, since no increase in mental distress was observed either for participants with normal test results nor for participants referred to PHCC. Most participants also reported low levels of worry about experiencing a myocardial infarction at baseline. That might reflect an expectation of normal findings and a perception of the health examination as a way to confirm their health, rather than discovering something [21].

There was an increase in mental distress among participants referred to the hospital and diagnosed with coronary artery stenosis, which indicates that one should be prepared in contexts like these for different risk outcomes and plan for support for those with higher risk. Our findings highlights that an increased utility of powerful, yet not fully mature, imaging techniques such as CT with coronary angiography requires careful considerations extending beyond medical risks and benefits; the clinician must also take into account the risk of mental distress and secure support when necessary. What would be a reasonable support for high risk patient is outside the scope of this study. However in our previous focus group study with SCAPIS participants, men who experienced worry after being referred to the hospital due to coronary artery stenosis, were calmed and felt safe after talking with the cardiologist and by taking preventive action [22].

Specifically worry has been associated with intention to take preventive action [23, 24]. A degree of specific worry might therefore not be something to avoid, but rather something that may help participants engage in preventive behaviors. Therefore it is a good idea to include options on self-management and risk reducing activities in risk communication to high risk individuals, factors that are also related to better health outcomes [25].

When comparing the results of this study to previous research, it is important to give consideration to the fact that the measurements for assessing psychological factors vary significantly between different studies, along with the type of psychological factor being measured. Neither we nor Nielsen et al observed an increase in depression after screening for coronary artery calcification [11]. While, an increase in anxiety was detected both in this study, as well as in others [5, 6]. Lokkegaard *et al*. detected no increase in psychotropic medication due to psychiatric diagnoses after screening for CV risk factors [9]. In our study, the increase in anxiety was significant, but considered small from a clinical perspective as it does not exceed the normal range and would not lead to a need for treatment of participants.

Another thing to consider is the difference between studies regarding whether or not an intervention was offered to participants, since the degree of support after returning an abnormal test result could potentially influence the impact of negative consequences. Jorgensen *et al*. did not detect an increase in anxiety [26] and in their study, both individual- and group counselling were part of the intervention. The participants in SCAPIS received no intervention except for routine care in the event of abnormal findings, making the result of this study comparable and relevant to CV risk assessment in regular health care settings with regularly offered health checks where the same conditions are often prevalent, as well as to ordinary health examinations offered to healthy individuals, often during the life span above 50 years of age.

### 4.1 Study limitations and strengths

One limitation of this study was the lack of a control group. The current design only allowed analyses of the differences among individuals who were referred to the hospital, to PHCC, or who were not referred at all. It is therefore unclear whether participation in SCAPIS itself caused any form of psychological distress. There was only one follow-up measurement three months after the participants’ first visits. Based on other studies a three months follow-up period seems appropriate for detecting an increase in mental distress after receiving risk information [27]. With an additional number of measure-points it would have been possible to know the extent to which the increased distress was transitory.

One strength of this study is the fact that participants were randomly selected. The sample was representative of Uppsala Municipality, except for a slightly smaller percentage of individuals who had only finished primary school [28]. The follow-up response rate was considered high (70%), but the follow-up sample had fewer participants with lower levels of education. There is also the potential that the individuals most affected by the disclosure of the results were lost to follow-up.

## Conclusion

Accordingly to our results, CV risk information can be provided to individuals with lower levels of risk without concerns of inducing mental distress. However, in order to prevent unnecessary worry in contexts similar to this study, one should be prepared for different risk outcomes and plan for support for individuals with higher risk. Furthermore, the increased utility of powerful, yet not fully mature, imaging techniques requires careful considerations extending beyond medical risks and benefits; the clinician must also take into account the risk of mental distress and secure support when necessary.
